# Effects of vegetation densities on the performance of attractive targeted sugar baits (ATSBs) for malaria vector control: a semi-field study

**DOI:** 10.1186/s12936-023-04625-z

**Published:** 2023-06-21

**Authors:** Letus L. Muyaga, Felician C. Meza, Najat F. Kahamba, Rukiyah M. Njalambaha, Betwel J. Msugupakulya, Emmanuel W. Kaindoa, Halfan S. Ngowo, Fredros O. Okumu

**Affiliations:** 1 Department of Environmental Health, and Ecological Science, Ifakara Health Institute, Morogoro, United Republic of Tanzania; 2 School of Biodiversity, One Health & Veterinary Medicine, University of Glasgow, Glasgow, UK; 3 Department of Vector Biology, Liverpool School of Tropical Medicine, Liverpool, UK; 4 School of Life Sciences and Biotechnology, Nelson Mandela African Institution of Science and Technology, Arusha, United Republic of Tanzania; 5 School of Public Health, University of the Witwatersrand, Johannesburg, South Africa; 6 Faculty of Health Sciences, School of Pathology, Centre for Emerging Zoonotic and Parasitic Diseases, Wits Research Institute for Malaria, University of the Witwatersrand, National Institute for Communicable Diseases, Johannesburg, South Africa

**Keywords:** *Anopheles arabiensis*, Attractive targeted sugar baits, ATSBs, Vegetation densities, Ifakara Health Institute, Outdoor biting, Indoor biting and semi-field

## Abstract

**Background:**

Attractive targeted sugar baits (ATSBs) control sugar-feeding mosquitoes with oral toxicants, and may effectively complement core malaria interventions, such as insecticide-treated nets even where pyrethroid-resistance is widespread. The technology is particularly efficacious in arid and semi-arid areas. However, their performance remains poorly-understood in tropical areas with year-round malaria transmission, and where the abundant vegetation constitutes competitive sugar sources for mosquitoes. This study compared the efficacies of ATSBs (active ingredient: 2% boric acid) in controlled settings with different vegetation densities.

**Methods:**

Potted mosquito-friendly plants were introduced inside semi-field chambers (9.6 m by 9.6 m) to simulate densely-vegetated, sparsely-vegetated, and bare sites without any vegetation (two chambers/category). All chambers had volunteer-occupied huts. Laboratory-reared *Anopheles arabiensis* were released nightly (200/chamber) and host-seeking females recaptured using human landing catches outdoors (8.00 p.m.–9.00 p.m.) and CDC-light traps indoors (9.00 p.m.–6.00 a.m.). Additionally, resting mosquitoes were collected indoors and outdoors each morning using Prokopack aspirators. The experiments included a “before-and-after” set-up (with pre-ATSBs, ATSBs and post-ATSBs phases per chamber), and a “treatment vs. control” set-up (where similar chambers had ATSBs or no ATSBs). The experiments lasted 84 trap-nights.

**Results:**

In the initial tests when all chambers had no vegetation, the ATSBs reduced outdoor-biting by 69.7%, indoor-biting by 79.8% and resting mosquitoes by 92.8%. In tests evaluating impact of vegetation, the efficacy of ATSBs against host-seeking mosquitoes was high in bare chambers (outdoors: 64.1% reduction; indoors: 46.8%) but modest or low in sparsely-vegetated (outdoors: 34.5%; indoors: 26.2%) and densely-vegetated chambers (outdoors: 25.4%; indoors: 16.1%). Against resting mosquitoes, the ATSBs performed modestly across settings (non-vegetated chambers: 37.5% outdoors and 38.7% indoors; sparsely-vegetated: 42.9% outdoors and 37.5% indoors; densely-vegetated: 45.5% outdoors and 37.5% indoors). Vegetation significantly reduced the ATSBs efficacies against outdoor-biting and indoor-biting mosquitoes but not resting mosquitoes.

**Conclusion:**

While vegetation can influence the performance of ATSBs, the devices remain modestly efficacious in both sparsely-vegetated and densely-vegetated settings. Higher efficacies may occur in places with minimal or completely no vegetation, but such environments are naturally unlikely to sustain *Anopheles* populations or malaria transmission in the first place. Field studies therefore remain necessary to validate the efficacies of ATSBs in the tropics.

**Supplementary Information:**

The online version contains supplementary material available at 10.1186/s12936-023-04625-z.

## Background

The scale-up of effective vector control tools, notably insecticide-treated nets (ITNs) and indoor residual spraying (IRS), coupled with effective case management and other measures have contributed to significant reductions of malaria burden in sub-Saharan Africa [1, 2]. Unfortunately, malaria control appears to be stagnating as many high-burden countries are reporting increased cases and deaths [3]. Many malaria programmes are recording diminishing returns amid a growing set of challenges, such as insecticide resistance, drug resistance and high commodity prices [4].

For vector control, there are also concerns of behavioral adaptations of mosquitoes, such as outdoor-biting and day-time biting [4–6]. These mosquito behaviours can overlap with human behaviours and activities to create new prevention gaps not effectively targetable with ITNs and IRS [7–10]. Continued progress towards malaria elimination in Africa, therefore, requires fundamental changes to current strategies, backed by improved financing and resource allocation as well as enhanced research into new transformative technologies [4, 11]. In particular, there is an urgent need for tools that remain effective against insecticide-resistant, outdoor-biting and day-time biting mosquitoes [12].

Attractive targeted sugar baits (ATSBs) are a promising new technology that is increasingly being investigated for expanding the malaria prevention toolbox [13–17]. The technology exploits the natural sugar-feeding behaviours essential for mosquito survival [18–22]. This means sugar-based solutions can be used to target mosquitoes by adding orally-ingested toxicants, which may include common pesticides, boric acid, and nucleic acids [23–28]. Other candidate toxicants include mosquitocidal drugs, such as ivermectin [29]. With any of the toxicants, ATSBs effectively target sugar-seeking male and female mosquitoes indoors or outdoors, thereby complementing the current primary interventions, such as ITNs even in areas where malaria vectors are resistant to pyrethroids [13]. The ATSBs could also be used to attract mosquitoes in order to feed them with peptides, drugs or micro-organisms that can block the development of parasites or viruses within mosquitoes to achieve paratransgenesis or refractoriness against pathogens [30, 31].

ATSBs can be deployed in different ways, e.g. by spraying on vegetation [15, 25] or suspending inside dwellings and around eave spaces [32]. However, the dominant and most scalable approach currently appears to be specially-designed membranes affixed to vertical surfaces such as outside walls of dwellings. In a large field trial in Mali, such ATSBs were particularly effective against older females of the malaria vectors and significantly impacted on malaria transmission [16]. In that trial, the proportion of females that had undergone at least three gonotrophic cycles was reduced by 97–100%; while the number of sporozoite-infected females was reduced by ~ 98% in the intervention villages compared to control villages [16].

Available evidence suggests that current ATSBs have generally been highly efficacious in arid and semi-arid regions [15, 16, 33]. However, there are doubts whether they would remain equally efficacious in tropical areas with year-round transmission, and where the abundant vegetation might constitute highly competitive sugar sources for mosquitoes. Given the current efforts to develop tools against outdoor-biting risk [12, 34], this concern should be investigated to identify critical points of improvement and to evaluate the performance of the ATSBs in the tropics. The aim of this current study was therefore to assess and compare the efficacy of outdoor ATSBs in settings with varying vegetation densities so as to inform further development, field-testing and deployment of ATSBs in different ecological systems in sub-Saharan Africa.

## Methods

### Semi-field systems

**Fig. 1:**
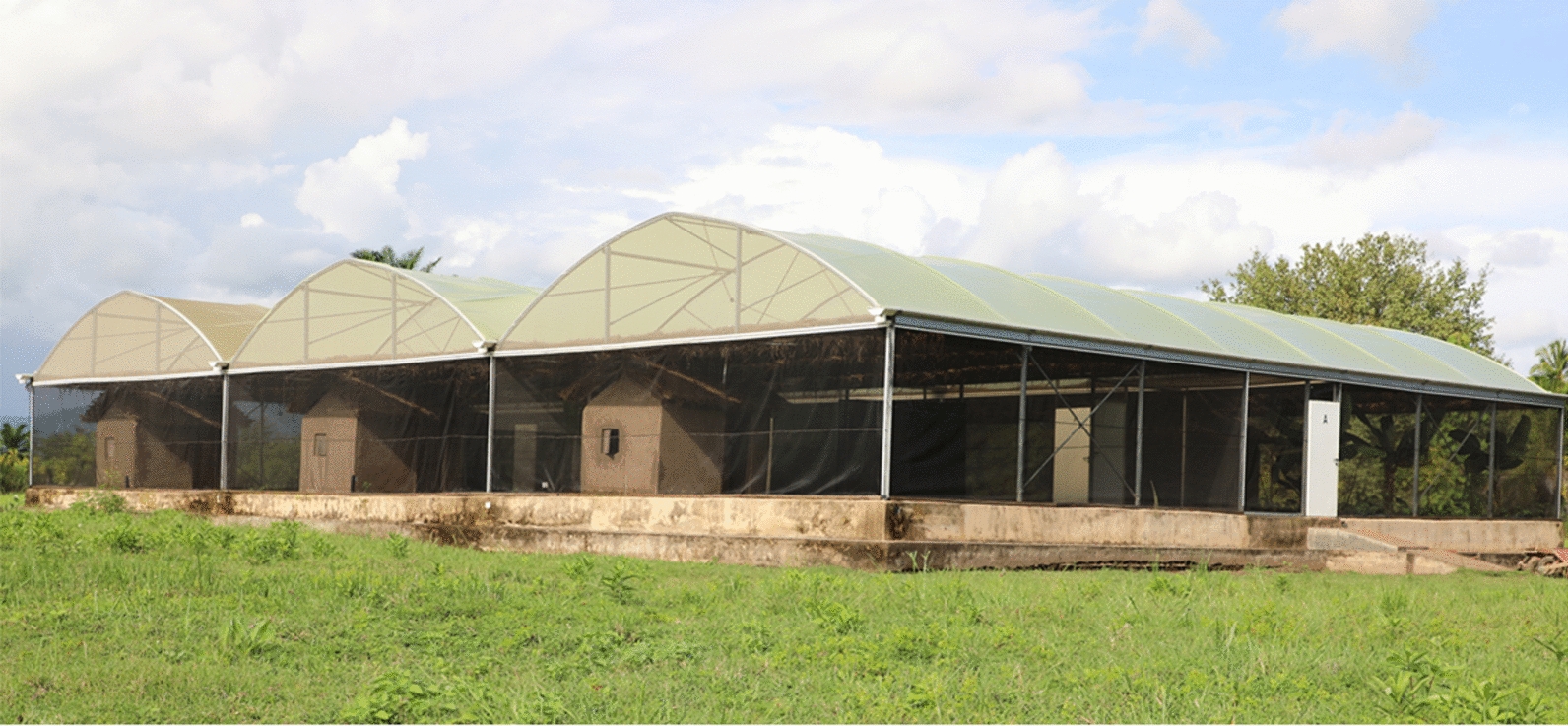
The semi-field system in which the experiments were conducted

This study was conducted inside the semi-field chambers at the Mosquito City facility of Ifakara Health Institute, in south-eastern Tanzania (8.10800° S, 36.66585° E) [35, 36]. Six chambers were used (Fig. 1). Since these enclosed systems experience high daytime temperatures, a layer of grass thatching was added to partially cover each chamber so as to prevent excess heat-related mortality of mosquitoes. Adjacent chambers were also separated using plastic sheetings to reduce the effects of microclimatic modification in one chamber from affecting the others.

### Plants

**Fig. 2:**
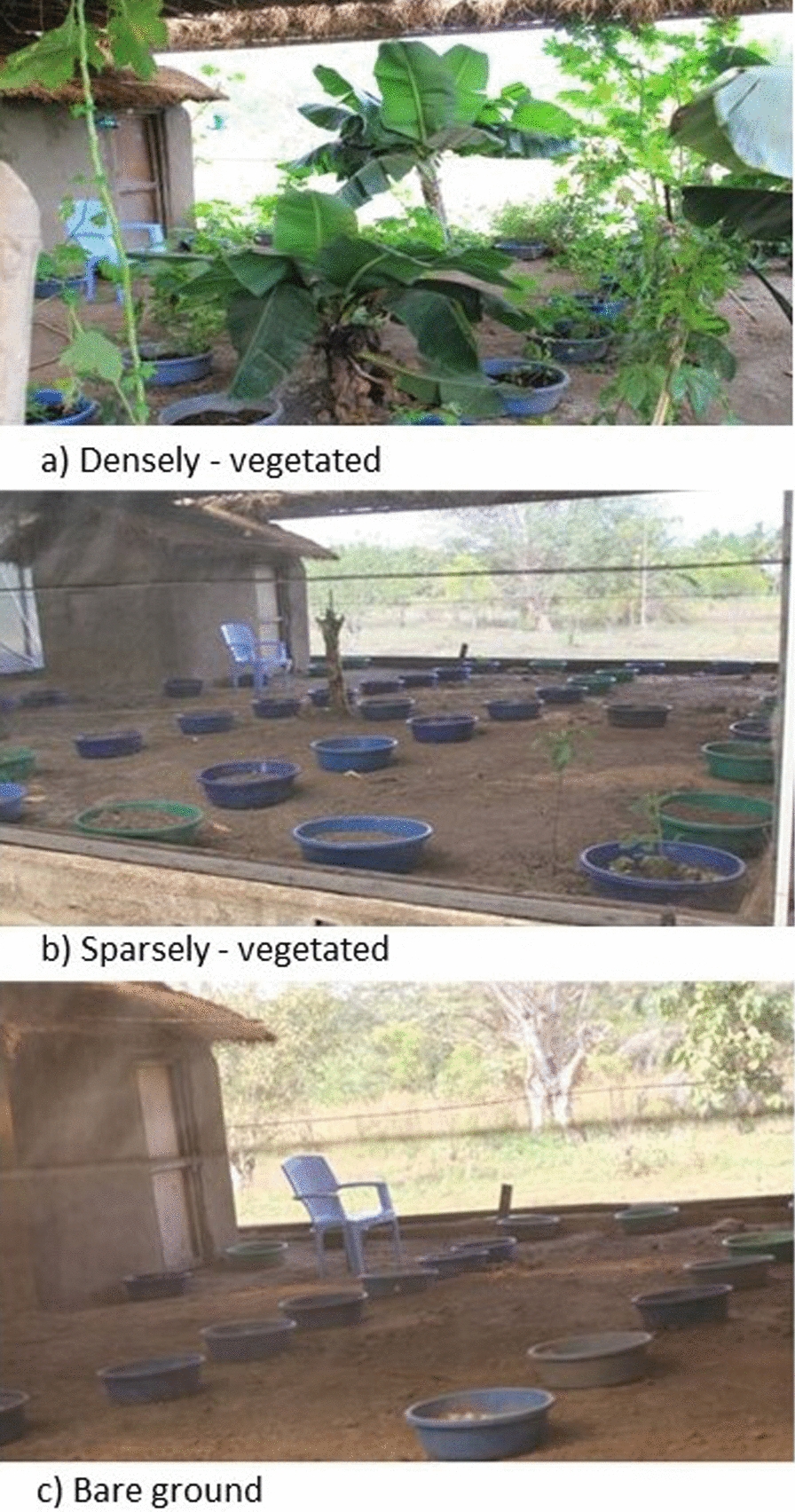
Vegetation densities in different semi-field chambers. Each of the densities, i.e. densely-vegetated, sparsely-vegetated and bare(no vegetation) environments were provided in duplicates (two chambers per category). In the bare chamber, soil-filled pots without plants were included so that the available resting surfaces for mosquitoes were similar in all chambers

Mosquito-friendly plants were identified based on published records of feeding and survival rates by malaria vectors [19]. Those that were available in the nearby areas of Ulanga and Kilombero districts, Tanzania, were selected for inclusion (Table 1). The selected plant species were potted and transplanted to the semi-field chambers, then allowed to flourish for at least 3 weeks before the trials began. Those that could not be potted were planted directly in the chambers (Fig. 2). Additionally, selected fresh fruits were sliced open and added at the start of the experiments as described below.

**Table 1:** Mosquito-friendly plants used for the experiment; selected based on published mosquito-preferences [19] and local availability in Ulanga and Kilombero districts, south-eastern Tanzania

Common name	Latin name	Mode of transplantation
Smooth pigweed	*Amaranthus hybridus* L.	Potted
Cocoyam	*Colocasia esculenta*	Planted directly in semi-field chamber
Castor bean	*Ricinus communis* L.	Potted
Sweet potatoes	*Ipomoea batatas* L.	Potted
Mexican fire plant	*Euphorbia heterophylla* L.	Potted
Plantain banana	*Musa paradisiacal*	Planted directly in semi-field chamber
Goat weed	*Ageratum conyzoides* L.	Potted
Mango tree	*Mangifera indica*	Planted directly in semi-field chamber
Guava tree	*Psidium guajava*	Planted directly in semi-field chamber
Watermelon fruit	*Citrullus lanatus*	Sliced fruit placed on pedestals

#### Vegetation densities in semi-field chambers

Two vegetation densities were considered. These included dense vegetation cover in two chambers and sparse vegetation cover in two other chambers. Two other chambers were left bare to simulate settings completely devoid of plants. To achieve the densities of densely-vegetated, sparsely-vegetated and bare chambers, the plants were provided in ratios of 50:5:0 for potted plants, 6:1:0 for directly transplanted vegetation, and 6:3:1 for cut fruit (Fig. 2). To ensure an equal resting surface for mosquitoes, the total number of pots was maintained at 50 per chamber, but those in the bare chamber contained only soil and no plants.

### Attractive targeted sugar baits (ATSBs)

**Fig. 3:**
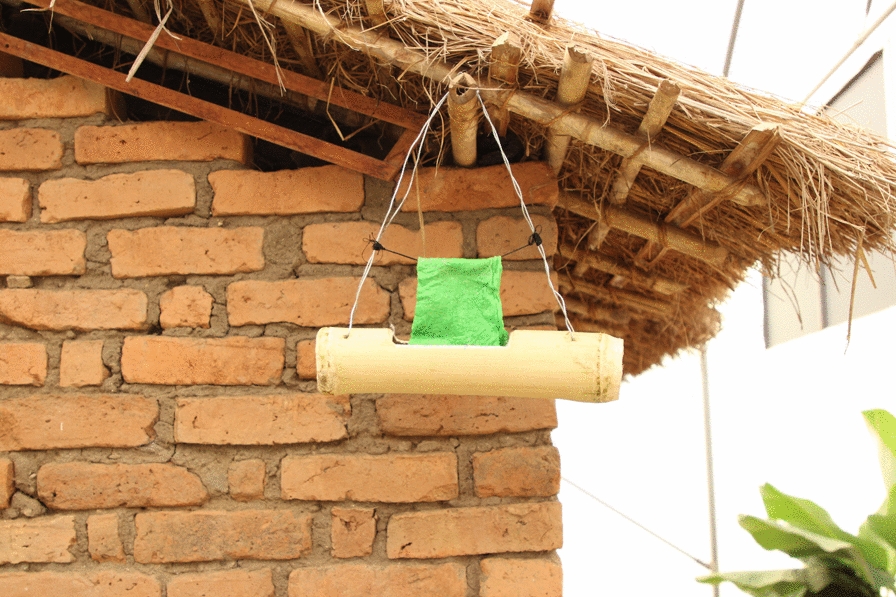
Pictorial representation of the ATSBs used in this experiment. The image also shows how the baits were positioned near the experimental huts in the semi-field chambers

The candidate ATSBs contained 2% boric acid (active ingredient) prepared in a 10% brown sugar solution. A green food dye was added to enable visual identification of mosquitoes that fed on the ATSBs. Preliminary tests comparing different ATSBs before selecting the test product are provided in Additional file 1. The ATSBs were prepared in locally-made receptacles made of laterally-sliced bamboo stems (Fig. 3). The ATSB solution was poured into these sliced containers, and cotton wool soaked into it. Five bait stations were placed around the hut to maximize the feeding success, this configuration was done by slightly adapting configurations described in Mali by Diarra et al. [37].

### Mosquitoes

Laboratory-reared 3–5 day old nulliparous adult females of *Anopheles arabiensis* were used. The mosquitoes were aspirated into small cages and starved for 9 h before being released. The mosquitoes were obtained from a common insectary maintained within the vector biology laboratory, the VectorSphere, at Ifakara Health Institute. This insectary is maintained under controlled conditions of 25–27 °C and 80% relative humidity. The larvae were fed on Tetramin® fish food and adults provided with a 10% sugar solution. A total of 200 mosquitoes were released in each chamber each evening at 6 p.m. and allowed to acclimatize in the environment for 2 h prior to the start of any recapturing. This was done in all chambers, regardless of the presence or absence of ATSBs, by gently opening and shaking the release cages so that no mosquito remained in the cages.

### Mosquito collections

In all tests, the process of recapturing host-seeking mosquitoes began 2 h after the starved mosquitoes had been released into the chambers. Human landing catches (HLC) were used to recapture outdoor-biting mosquitoes from 8.00 p.m. to 9.00 p.m. each night, after which the volunteers entered the huts inside each chamber to sleep under intact untreated nets. Thereafter, CDC light traps set beside the volunteer-occupied bed net was used to collect indoor host-seeking mosquitoes from 9.00 p.m. to 6.00 a.m. the following morning. Each morning at 6.00 a.m., Prokopack aspirators were used to collect resting mosquitoes inside and outside the huts for a fixed 20 min each morning. At the end of each test night, the Prokopack aspirators were used to remove any mosquitoes remaining in the chambers as exhaustively as possible, as the chambers prepared for the next trap-night.

These mosquito collection procedures were maintained on all experimental nights and in all chambers regardless of presence or absence of ATSBs. To avoid biases in host-attractiveness, the volunteers were rotated between the experimental chambers each night. Each morning, all the recaptured mosquitoes (by HLC, CDC-light traps and Prokopack aspirators) were killed and their abdomen examined for evidence of having fed on ATSBs. In the initial tests, all the mosquitoes that had fed on the ATSBs died within 24 h (Additional file 1). Therefore all recaptured mosquitoes with ATSB markers in their abdomen were considered killed and out of circulation, thus included as dead.

### Evaluating the efficacy of ATSBs

An initial test was conducted to evaluate the performance of ATSBs and to confirm their suitability for the main study. This experiment involved three chambers without vegetation and was conducted in two phases lasting a total of 24 consecutive nights, on a “before-and-after” set up. In the first phase, no ATSBs were used (16 nights; pre-intervention period), while in the second phase, the ATSBs were introduced in the same chambers (8 nights; intervention period).

The number of mosquitoes trapped by HLC (outdoor-biting densities), CDC-light traps (indoor-biting densities), Prokopack indoors (indoor-resting densities), and Prokopack outdoors (outdoor-resting densities) were compared between pre-intervention period (no ATSBs) and the intervention period (ATSBs added) to assess the efficacy of the ATSBs in each chamber.

### Comparing the efficacy of ATSBs in chambers with dense, sparse and no vegetation

**Fig. 4:**
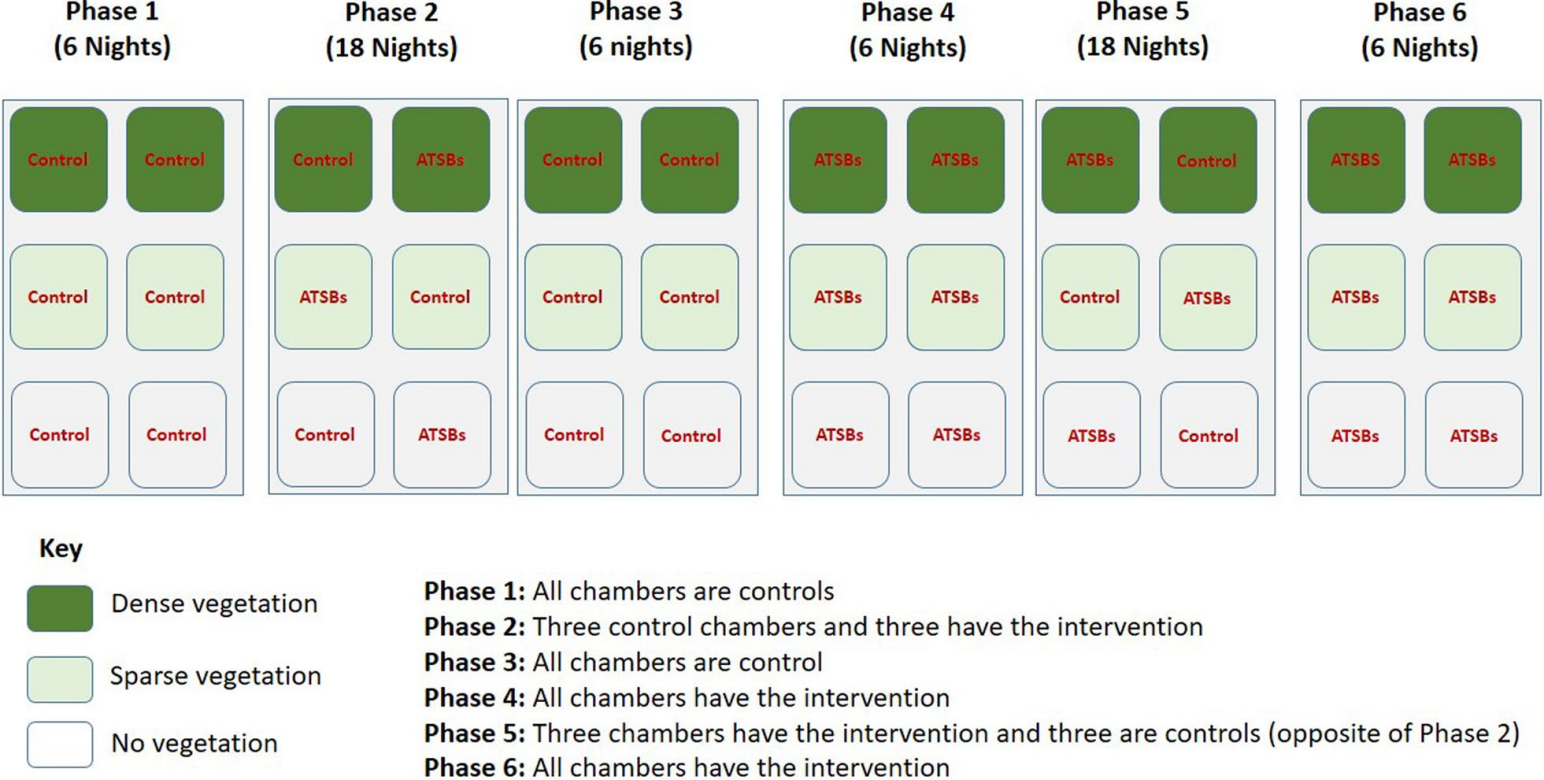
Assignment procedures for ATSBs in the chambers during tests to compare the performance of ATSBs in densely-vegetated, sparsely-vegetated, and bare (no vegetation) environments

Six semi-field chambers were used, two with dense vegetation, two with sparse vegetation, and another two with no vegetation (Fig. 2). The experiment was conducted for 60 nights in six phases as illustrated in Fig. 4.

The first phase (pre-intervention phase) involved mosquito collections as described above but without any ATSBs in the chambers. This phase lasted 6 consecutive nights. In the second phase, ATSBs containing 2% borate were introduced into one of the two densely-vegetated chambers, one of the two sparsely vegetated chambers and one of the two bare chambers (five ATSBs per chamber, each suspended around the eave space as shown in Fig. 3). The other chambers were left without ATSBs to constitute contemporaneous controls (one densely-vegetated chamber, one sparsely vegetated chamber and one bare chamber). Mosquito collections with HLC, CDC light traps and Prokopack aspirators continued as above for 18 consecutive nights. The third phase was identical to the first phase since the ATSBs were removed from all chambers, and the mosquito collections continued for another six nights. In the fourth phase, for another six nights, ATSBs were added in all chambers so that there were no contemporaneous controls. A fifth phase was conducted for 18 nights consecutively and was the reverse of the second phase in that the chambers that had been assigned ATSBs became controls while those that had been controls now received ATSBs. In the final phase (sixth phase), the ATSBs were returned to all the chambers and the experiment continued for another six nights consecutively.

Based on this configuration, there were therefore 12 nights during which all the chambers were controls, 12 nights during which all the chambers had ATSBs and 36 nights during which half of the chambers (one densely-vegetated, one sparsely vegetated, and one bare) were controls while the other half had ATSBs. Therefore, in addition to assessing the impact of vegetation cover, the design also enabled valuation of the effects of introducing ATSBs into chambers that previously had none, as well as comparing similar chambers with and without the ATSBs.

### Statistical analysis

Data analysis was done using the open-source statistical software, R version 3.6.2 [38]. The overall impact of ATSBs in chambers with different vegetation densities and chambers without vegetation was examined using data from each trapping method. This was done to understand effects on host-seeking mosquitoes outdoors (HLC catches), host-seeking mosquitoes indoors (CDC-Light trap catches), indoor-resting mosquitoes (Prokopack trap collections inside the huts) and outdoor-resting mosquitoes (Prokopack trap collections outside the huts). The analysis was done using generalized linear mixed effects models (GLMM), implemented using the *lme4* package [39], following a binomial distribution. To assess effects of ATSBs, the number of mosquitoes recaptured was added as a response variable, while the intervention (ATSBs or no ATSBs) was added as a fixed factors. Volunteers ID, chamber ID, and experimental day were added as random terms in the models.

The efficacy of ATSBs was calculated as $Efficacy=\frac{Control-Treatment}{Control}*100$, where “Control” was the number of mosquitoes recaptured in absence of ATSBs, and “Treatment” was number of mosquitoes recaptured in presence of ATSBs.

A separate set of GLMM models were used to compare performance of the ATSBs under different vegetation covers by considering data in the contemporaneous treatment and control chambers. Mosquito catches were used as response variable, while vegetation densities were added as fixed variable. Again, to account for any sampling biases and any unexplained variations between chambers and days, volunteers ID, chamber ID, and experimental day were added as random terms. Odds ratios and respective 95% confidence intervals were reported.

All graphs were created using the *ggplot2* package in R [40]. Lastly, Chi-Square test of proportions was used to assess whether there was any significant difference between the percentage reduction of mosquito catches in densely-vegetated chambers compared to sparsely-vegetated chambers and chambers devoid of any vegetation.

Model selection for the inclusion of the random effects (volunteers ID, chamber ID, and experimental day) was done using Akaike Information Criterion (AIC), the model with the lowest AIC value was considered as a best model.

## Results

### Efficacy of ATSBs

**Fig. 5:**
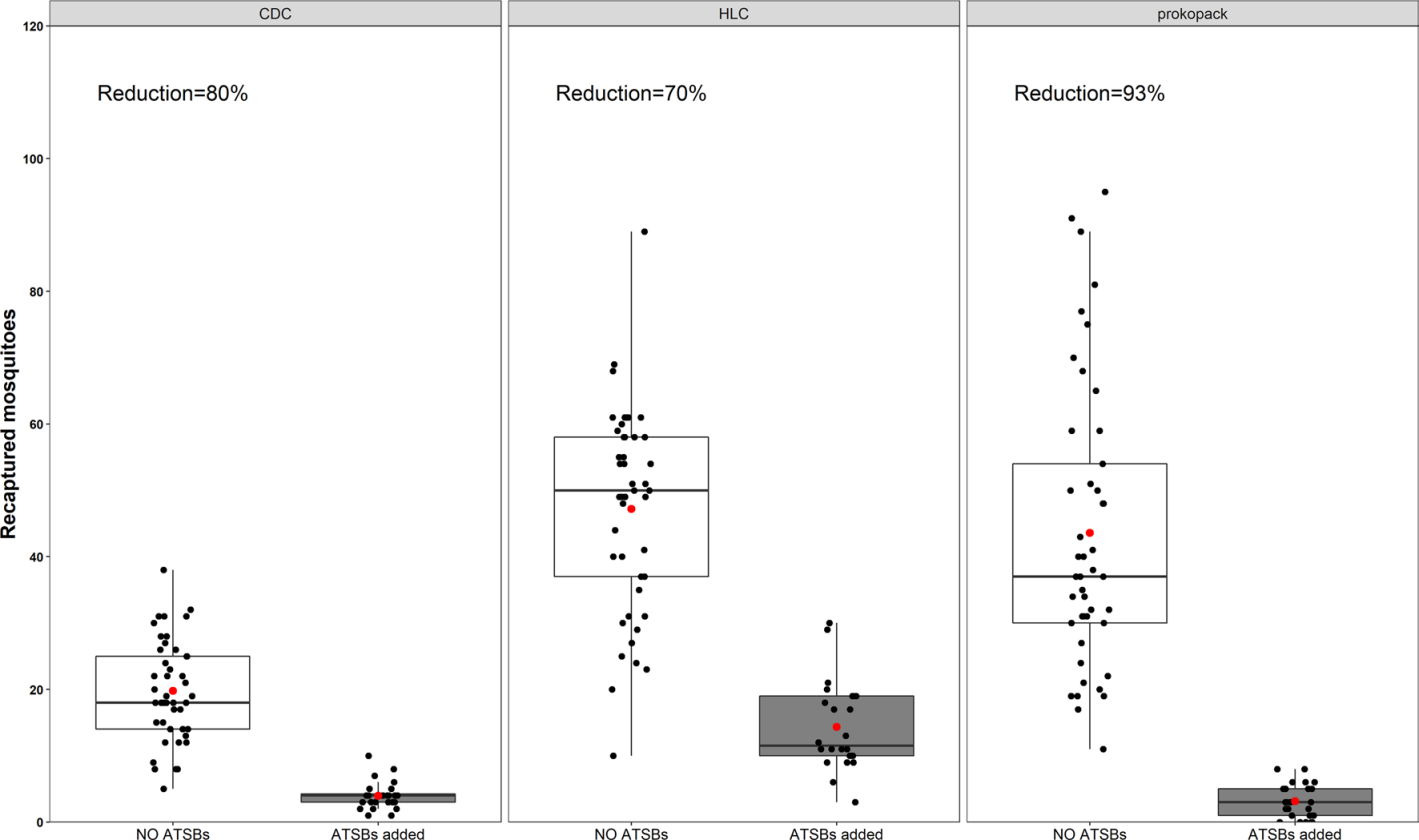
Performance of the attractive targeted sugar bait (ATSBs), when tested in bare chambers devoid of any vegetation. The Figures show the number of mosquitoes recaptured with CDC light traps, human landing catches (HLC) and Prokopack aspirators with or without ATSBs. The red dots represents the mean and the black dots represents daily collections

In the initial tests conducted to assess baseline performance of the ATSBs in bare un-vegetated chambers, introduction of the baits reduced the number of outdoor-biting *An. arabiensis* females (as measured using HLC) by 69.7% (95% CI 68.3–71.0%). In the same tests, the indoor-biting densities (as measured using CDC light traps) were reduced by 79.8% (78.9–81.1%) and the overall resting densities indoors and outdoors by 92.8% (91.6–94.6%). Full details of the findings are summarized in Table 2; Fig. 5.

**Table 2:** Performance of the candidate attractive targeted sugar bait (ATSB) when tested in bare chambers without vegetation

Parameter estimated	Trap used	Intervention	Mean (95% CI)	% Reduction	OR 95% CI	P-value
Indoor-biting risk	CDC light traps	No ATSBs	19.8 (18.6–20.9)	79.8 (78.9–81.1)	1	< 0.001
		ATSBs added	4.0 (3.5–4.4)		5.4(3.8–7.6)	
Outdoor-biting risk	Human landing catches (HLC)	No ATSBs	47.2 (44.9–49.5)	69.7 (68.3–71.0)	1	< 0.001
		ATSBs added	14.3 (13.0–15.7)		3.9(2.8–5.5)	
Resting densities (indoors and outdoors)	Prokopack aspirators	No ATSBs	43.6 (37.1–50.1)	92.8 (91.6–94.6)	1	< 0.001
		ATSBs added	3.1 (2.0–4.2)		17.8(10.6–29.9)	

### Efficacy of ATSBs in semi-field chambers with dense, sparse and no vegetation

**Fig. 6:**
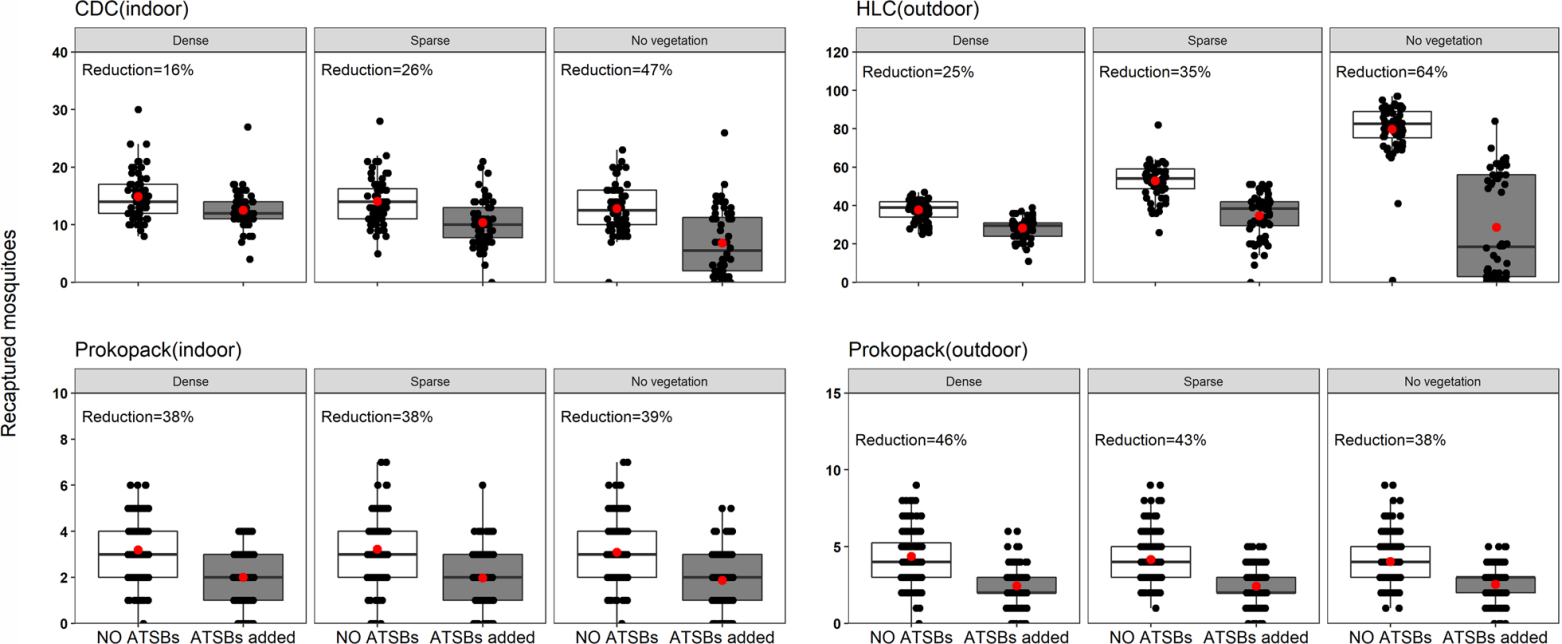
Comparative performance of the attractive targeted sugar baits (ATSBs) in chambers with dense, sparse, or bare (no vegetation). Figures show the number of mosquitoes recaptured with CDC light traps, human landing catches (HLC) and Prokopack aspirators with or without ATSBs. The red dots represent the mean and the black dots represents daily collections

The introduction of the ATSBs significantly reduced the number of female *An. arabiensis* mosquitoes attempting to bite volunteers outdoors in all chambers, but mostly in the chambers with no vegetation. The catches were reduced by 25.4% (95% CI 24.2–26.6%) in densely-vegetated chambers by 34.5% (31.5–37.6%) in sparsely-vegetated chambers and by 64.1% (57.0–71.2%) in bare chambers without any vegetation (Fig. 6; Table 3). Chi-square tests conducted on the outdoor-biting data showed a significant difference in the impacts of ATSBs between chambers with the different vegetation densities (χ^2^ = 346.5, p < 0.001), suggesting that vegetation cover reduced the efficacies of ATSBs against mosquitoes biting outdoors (Table 4).

**Table 3:** Effect of candidate ATSBs in semi-field chambers with dense, sparse and no vegetation

Vegetation densities	Parameter estimated	Intervention	Mean mosquito catches (95% CI)	Reduction (%)	OR (95% CI)	P-value
Dense vegetation	Indoor-biting risk	No ATSBs	14.9 (13.8–16.0)	16.1 (15.9–16.9)	1	< 0.001
		ATSBs added	12.5 (11.6–13.3)		1.2 (1.1–1.3)	
	Outdoor-biting risk	No ATSBs	37.8 (36.4–39.2)	25.4 (24.2–26.6)	1	< 0.001
		ATSBs added	28.2 (26.7–29.7)		1.4 (1.3–1.6)	
	Indoor-resting	No ATSBs	3.2 (3.1–3.3)	37.5 (36–38.7)	1	< 0.001
		ATSBs added	2.0 (1.9–2.1)		1.6 (1.4–1.8)	
	Outdoor-resting	No ATSBs	4.4 (4.1–4.6)	45.5 (43.9–45)	1	< 0.001
		ATSBs added	2.4 (2.3–2.6)		1.8 (1.6–2.1)	
Sparse vegetation	Indoor-biting risk	No ATSBs	14.1 (13.0–15.1)	26.2 (24.5–28.5)	1	< 0.001
		ATSBs added	10.4 (9.3–11.4)		1.4 (1.2–1.6)	
	Outdoor-biting risk	No ATSBs	52.8 (50.5–55.2)	34.5 (31.5–37.6)	1	< 0.001
		ATSBs added	34.6 (31.5–37.8)		1.9 (1.7–2.0)	
	Indoor-resting	No ATSBs	3.2 (3.1–3.3)	37.5 (36.4–38.7)	1	< 0.001
		ATSBs added	2.0 (1.9–2.1)		1.7 (1.4 -1. 9)	
	Outdoor-resting	No ATSBs	4.2 (3.9–4.4)	42.9 (40.9–43.6)	1	0.71
		ATSBs added	2.4 (2.2–2.6)		1.7 (1.5–1.9)	
No vegetation	Indoor-biting risk	No ATSBs	12.8 (11.8–13.9)	46.8 (39.7–55.1)	1	< 0.001
		ATSBs added	6.8 (5.3–8.4)		2.1 (1.8–2.4)	
	Outdoor-biting risk	No ATSBs	79.7 (76.0–83.4)	64.1 (57.0–71.2)	1	< 0.001
		ATSBs added	28.6 (21.4–35.9)		5.0 (4.6–5.4)	
	Indoor-resting	No ATSBs	3.1 (2.9–3.3)	38.7 (36.4–41.2)	1	< 0.001
		ATSBs added	1.9 (1.7–2.1)		1.7 (1.4–1.9)	
	Outdoor-resting	No ATSBs	4.0 (3.9–4.1)	37.5 (36.7–38.5)	1	< 0.001
		ATSBs added	2.5 (2.4–2.6)		1.6 (1.4–1.8)

**Table 4:** Effects of vegetation densities on performance of the ATSBs (when the observations in chambers with no vegetation are considered as reference). Comparative effects of the vegetation densities are estimated by Chi-Square test

Parameter estimated	Trap	Vegetation cover	Mean (± 2SE)	OR 95% CI	P-value	χ^2^, p-value
Indoor biting risk	CDC light traps	No vegetation	6.8 (5.3–8.4)	1		(33.12, < 0.001)
		Sparse vegetation	10.4 (9.3–11.4)	0.64 (0.56–0.74)	< 0.001	
		Dense vegetation	12.5 (11.6–13.3)	0.53 (0.47–0.60)	< 0.001	
Outdoor biting risk	Human landing catches (HLC)	No vegetation	28.2 (21.4–35.9)	1		(346.05, < 0.001)
		Sparse vegetation	34.6 (31.5–37.8)	0.79 (0.66–0.95)	0.01	
		Dense vegetation	28.2 (26.7–29.7)	1.02 (0.85–1.22)	0.87	
Indoor-resting densities	Prokopack aspirators (indoors)	No vegetation	1.9 (1.7–2.1)	1		(0.15, 0.93)
		Sparse vegetation	2.0 (1.8–2.1)	0.95 (0.81–1.11)	0.53	
		Dense vegetation	2.0 (1.8–2.2)	0.93 (0.8–1.09)	0.39	
Outdoor-resting densities	Prokopack aspirators (outdoors)	No vegetation	2.5 (2.4–2.7)	1		(1.97, 0.37)
		Sparse vegetation	2.4 (2.2–2.6)	1.05 (0.91–1.2)	0.51	
		Dense vegetation	2.4 (2.3–2.6)	1.04 (0.91–1.19)	0.58	

The number of female *An. arabiensis* mosquitoes caught attempting to bite the volunteer while sleeping under the bed net indoors (i.e. indoor-biting risk as measured by CDC light traps) were reduced by 16.1% (95% CI 15.9–16.9%) in densely-vegetated chambers, 26.2% (24.5–28.5%) in sparsely-vegetated chambers and 46.8% (39.7–55.1%) in bare chambers with no vegetation (Fig. 6; Table 3). Here also, the Chi-square analysis showed that there was a significant difference of the impact of ATSBs between chambers with different vegetation densities (χ^2^ = 33.12, p < 0.001), again suggesting that vegetation cover reduced the efficacies of ATSBs against mosquitoes biting indoors (Table 4).

Lastly, the ATSBs also reduced the densities of resting mosquitoes (collected using Prokopack aspirators). The outdoor-resting densities were reduced by 45.5% (95% CI 43.9–45.0%) in the densely-vegetated chambers, by 42.9% (40.9–43.6%) in the sparsely vegetated chambers, and 37.5% (36.7–38.5%) in the chambers without any vegetation. Data obtained from the HLC catches, appeared to show a bimodal distribution though the differences between intervention and control arms were still readily detectable (Fig. 6; Table 3). However there was no difference in the efficacy of the ATSBs between the chambers with different vegetation densities (χ^2^ = 1.97, p = 0.37). Similarly, the indoor-resting densities were reduced, in this case by 37.5% (95% CI 36.0–38.7%) in chambers with dense vegetation, 37.5% (36.4–38.7%) in chambers with sparse vegetation, and 38.7% (36.4–41.2%) in bare chambers without vegetation. Here too, there was no differences in impact between the chambers (χ^2^ = 0.15, p = 0.93) (see Table 4). Vegetation did not affect the efficacy of ATSBs against resting mosquitoes, either indoors or outdoors (Table 4). Overall, the total number of mosquitoes collected while resting was 3.1-fold lower than the numbers caught while host-seeking (i.e. by HLC and CDC-light traps).

## Discussion

Mosquitoes feed primarily on natural sugar sources like plant leaves, nectaries, and honeydew, a behaviour that can be exploited to target both their male and female adults. The use of ATSBs has increasingly been demonstrated to effectively control malaria vectors, mostly in dry and semi-arid regions [15, 25, 32]. The technology has the added advantage that it can be used against both indoor-biting and outdoor-biting mosquitoes, including those that are active during the day. Moreover, by relying on oral insecticides, it offers a different mode of activity compared to currently approved vector control insecticides, many of which act on contact with insect cuticle [41]. Given these attributes, ATSBs could be highly effective in addressing current limitations of core malaria interventions such as ITNs and IRS even in areas where mosquitoes are resistant to pyrethroids, the commonest pesticide class used on ITNs.

This current study investigated the concern that this technology may not be equally efficacious in highly-vegetated settings where there would be numerous natural sugar sources, potentially competing with the ATSBs for the same mosquitoes. The large screened cages available at the Ifakara Health Institute’s Mosquito City facility [35, 36] provided a functional mesocosm to evaluate the effects of vegetation densities and compare the performance of ATSBs under controlled environments. Such an objective would otherwise require geographically-extensive and expensive trials to accomplish. Whereas such semi-field systems do not fully replace natural settings, they can enable direct and rapid assessments of important mosquito life cycle traits such as blood-feeding, sugar-feeding, mating and resting behaviours [35, 42, 43], as well as genetic and phenotypic variabilities [44]. These systems can also enable controlled assessments of the performance of new and existing vector control interventions. In previous studies, they have been used to test ITNs, zooprophylaxis, and endectocides [36], spatial repellents [45] and mosquito-assisted larviciding [46] and eave tubes [47].

In this study, the semi-field systems were used to simulate areas with varying vegetation densities, i.e. densely-vegetated, sparsely-vegetated and bare grounds with no vegetation, to enable comparison of the efficacy of ATSBs. The main findings were as follows: (i) the efficacy of ATSBs against indoor or outdoor-biting mosquitoes was highest in areas devoid of any vegetation; (ii) while there was a significant effect of varying vegetation densities on the performance of ATSBs, the devices still provided modest protection in both densely-vegetated and sparsely-vegetated settings; and (iii) the vegetation-dependent differences in the efficacy of ATSBs were observed for host-seeking mosquitoes (indoors and outdoors) but not resting mosquitoes due to low recaptures, against which the ATSBs performed modestly regardless of vegetation cover. Collectively, these findings suggest that ATSBs may remain modestly efficacious even in tropical countries where there is moderate to dense vegetation.

Mathematical evaluations of the potential impact of ATSBs have previously shown that even with modest mortality rates imparted by ATSBs on mosquito populations, the overall impact on malaria infections may be substantial [48, 49]. The modest efficacies observed in this study may therefore be indicative of greater potential and should be validated in field trials in different settings; for example through observational or randomized controlled trials. The observed differential efficacies in vegetated compared to non-vegetated settings suggest that natural sugar sources could indeed compete with the ATSBs, though such competition is unlikely to render the ATSBs fully ineffective.

Published evidence suggests that the modest efficacies observed for ATSBs are not uncommon. In the large scale Mali trial by Traore et al., the application of ATSBs was associated with 26.3% reduction in human-biting densities as measured by HLC, even though the baits appeared to preferentially kill older mosquitoes [16]. This trial also indicated that reduction of densities as measured by other trap types did not exceed 60% during the wet season. In the sub-tropical environments of Florida, Qualls et al. [50] evaluated ATSBs in a location surrounded by pine forests, wetlands and large ponds and observed more than 50% lower densities of *Anopheles crucians* for more 3 weeks. Similarly, Müller et al., showed in a small oasis full of vegetation and small freshwater springs in a desert area that ATSBs reduced the catches of *Anopheles sergentii* by 39% [51]. Interestingly, far higher efficacies, such as those observed in the initial experiments in this current study (Table 3; Fig. 5), have also been observed in field studies. For example, Beier et al. [33] applied ATSBs in areas with sugar-poor and sugar-rich oases and demonstrated that availability of local plants could delay but not compromise the technology; in this case they reduced the densities of *An. sergentii* over 95%.

Another finding from this study was that the reduction in mosquito densities was highest in non-vegetated settings (Table 1), a finding potentially attributed to limited sugar sources [33]. However, the high performance of ATSBs in chambers devoid of any vegetation may not be of any practical implications since such environments would generally be unlikely to sustain natural *Anopheles* populations nor malaria transmission; thus they may not be priority settings for the deployment of ATSBs. In the field studies in Mali by Traore et al. [16], the populations reduction, despite being modest in the wet seasons, exceeded 70% during the dry season for both male and female mosquitoes. Given that plant life is lower in dry seasons than wet seasons, this finding partially matches our observations that ATSBs were most efficacious in non-vegetated followed by sparsely-vegetated then densely-vegetated settings.

It was also interesting to observe that the efficacy against resting mosquitoes was similar across all vegetation densities assessed, even though there had been clear differences when considering host-seeking mosquitoes. This lack of statistically-significant differences is partly due to the lower densities of resting mosquitoes collected compared to the densities caught by HLC and CDC light traps. Overall, there were fewer mosquitoes collected resting compared to those collected host-seeking indoors and outdoors. The observations of ATSBs retaining high efficacy despite competing plants and flowers have also previously been recorded in Mali in a semi arid region, where the relative abundance of both male and female *Anopheles gambiae* declined by 90% [15]. One question is whether the concentration of active ingredient can be varied to maintain high efficacies in highly-vegetated settings. Previous bioassays in mosquito cages, the density of *An. gambiae* killed by boric acid were observed to increase with increasing concentrations of boric acid [14]. Future studies should therefore also assess the possibility that any negative impacts of vegetation on the performance of ATSBs can be reversed by increasing the concentration of the active ingredient.

The application of ATSBs outdoors, as done in this study, appears to have had implications for both indoor and outdoor-densities of mosquitoes. Mosquito densities were also reduced indoors across all vegetation covers (Tables 2 and 3). In earlier studies by Müller et al. in Mali [25], where the toxic baits were deployed near larval habitats, the mosquito densities declined by up to 94% [25]. Similar impacts were observed in Tanzania where ATSBs were applied to places near rice fields [13]. Separately, in a field study in Mali covering fourteen villages, half of which received ATSBs outdoors, it was observed that mosquito densities indoors (measured by CDC light traps) also declined by 57% [49].

To the best of our knowledge, this is the first study to directly assess the impact of vegetation densities on the performance of ATSBs. While the objectives were broadly achieved, a number of limitations are noted. Notably, the vegetation categories and plant ratios (dense, sparse and bare) were broadly arbitrary and might not be fully representative of all malaria-endemic communities. Furthermore, the candidate plants used here were those which had been previously shown to be preferred by mosquitoes [19]. This study did not include any plants with repellent properties, which might also have had an influence on the efficacy of the ATSBs. Another limitation is that we used a fixed number of bait stations, given that increasing number of bait stations can increase the feeding rate [37], future studies and deployment may need to optimize the ratio of ATSBs/household or ATSBs for every given area to maximize potential.

There were some limitations regarding the methods of used. In particular, the vegetation densities were not rotated and instead this study combined a before-and-after design with a “intervention vs. control” comparison. The main reason for repeating the phases was to account for any variation caused by nightly weather changes, as random assignment in such a limited setting could potentially miss such variations and hinder the assessments of ATSBs efficacy. Besides, since some of the larger plants were actually planted in the chambers, rather than being potted, it was not possible to rotate the independent variable (vegetation densities). Instead, it became necessary to rotate the interventions between the chambers with similar vegetation covers. Moreover, since there were only six chambers available in total, the maximum number that could be assigned to any vegetation density was only two. To address these limitations, the study used multiple repeated measures in each semi-field chamber over a total of 84 nights, each time ensuring an appropriate set of controls. Broadly however, it should be noted that these systems remain limited and that such as study would be more realistic if they were: (a) conducted in real field settings in multiple villages having different vegetation densities over multiple seasons, (b) include a placebo treatment for the control arms, as well as appropriate blinding of volunteers to the study phases and interventions and (c) include enhanced statistical evaluations to better fit the data distribution, e.g. bimodal distributions in the HLC catches illustrated in Fig. 5.

Secondly, it is possible that ATSBs may affect the natural mosquito foraging behaviours, and change the proportions foraging outdoors or indoors. In this study, the ATSBs were located at the eaves level and may potentially have caused a shift between indoor to outdoor feeding proportions. Unfortunately, since there were no comparative trapping observations between the indoor and outdoor locations, it was not possible to evaluate such effects in this study. Lastly, since this study was conducted for only *An. arabiensis*, further studies might focus on other species, such as *Anopheles funestus*, *An. gambiae* and *Anopheles coluzzii*, which are also major vectors in Africa.

## Conclusion

Attractive targeted sugar baits control sugar-seeking mosquitoes with oral toxicants and can be used both indoors or outdoors. This study has demonstrated that while vegetation densities can indeed influence the performance of ATSBs, the technology is likely to be at least modestly efficacious in sites with varying vegetation densities including sparsely-vegetated and densely-vegetated settings. It is likely that higher efficacies could be achieved in places with completely no vegetation, but such settings are naturally unlikely to sustain *Anopheles* mosquitoes nor malaria transmission locally. Additional field studies therefore remain necessary to validate performance of ATSBs in different settings in the tropics. Such studies should also consider investigating the efficacy ATSBs applied indoors and outdoors.

## Supplementary Information

**Additional file 1.** A additional file to provide details on preliminary studies done prior to and in support of the main study.

## Data Availability

Data are available at Ifakara Health Institute data for sharing upon request.

## Abbreviations

ATSBs: Attractive targeted sugar baits
IHI: Ifakara Health Institute
IRB: Institutional Review Board
ITNs: Insecticide Treated Bed Nets
IMR: National Institute for Medical Research
CDC: Centre for disease control and prevention
HLC: Human landing catch
IRS: Indoor residual spraying
