# Supplementary material for: Effects of vegetation densities on the performance of attractive targeted sugar baits (ATSBs) for malaria vector control: a semi-field study
Source: Malar J. 2023 Jun 21;22:190. doi: 10.1186/s12936-023-04625-z (PMC10283285; doi:10.1186/s12936-023-04625-z)
Supplement: Supplementary file 1 — Additional file 1. A additional file to provide details on preliminary studies done prior to and in support of the main study. [file 12936_2023_4625_MOESM1_ESM.docx]

**Effects of vegetation densities on the performance of attractive targeted sugar baits (ATSBs) for malaria vector control: a semi-field study**

Letus L. Muyaga^1^*, Felician C. Meza^1^, Najat F. Kahamba^1,2^,Rukiyah M. Njalambaha^1^ , Betwel J. Msugupakulya^1,3^, Emmanuel W. Kaindoa^1,4,6^, Halfan S. Ngowo^1,2^ and Fredros O. Okumu^1,2,4,5^*

**Background**

This is a Additional file to provide details on preliminary studies done prior to and in support of the main study. These studies informed the decision of the final protocol and study design of the main study, which was to compare the efficacy of ATSBs in settings with different vegetation densities. The supplementary studies included in this Additional file involved three different experiments as follows.

1. **Experiment 1:** Baseline laboratory evaluation of the efficacy of ATSBs with 1%, 2% and 3% boric acid (active ingredient)
2. **Experiment 2:** Evaluation of the efficacy of ATSBs (active ingredient: 1% boric acid) in densely-vegetated (dense), moderately vegetated (medium), and sparsely-vegetated (sparse) chambers. After completion of these studies, a decision was made to use only dense and sparse vegetation densities, since there appeared to be no difference between the medium and dense vegetation densities
3. **Experiment 3:** Evaluation of the efficacy of ATSBs in densely-vegetated, sparsely-vegetated and bare chambers using 1% of boric acid.

**Experiment 1: Laboratory evaluation of the efficacy of ATSBs (cage bioassays)**

This study was conducted in small cages for three replicates. Three different concentration (1%, 2% and 3% boric acid) were evaluated. A cage without ATSBs was used as a control for each replicate. Feeding mortality rates were recorded

The results were as follows: Percentage mortality was 81% with 1% a.i, 93% with 2% a.i and 96% with 3% a.i (Table S1).

**Table S1**: Efficacy of ATSBs with 1%, 2% and 3% boric acid against laboratory-reared *An. arabiensis* mosquitoes

| Concentration of boric acid in the ATSBs (%) | Proportion of mosquitoes that fed on the ATSBs | Percentage mortality of mosquitoes 24hrs after exposure to the ATSBs |
| --- | --- | --- |
| 0% | 93 % | 8 % |
| 1% | 81 % | 100 % |
| 2% | 93 % | 100 % |
| 3% | 96 % | 100% |

**Experiment 2: Comparative evaluation of the efficacy of ATSBs with 1% boric acid in chambers with dense, medium and sparse vegetation covers.**

Six semi-field chambers were used, two with dense vegetation, two with medium vegetation, and another two with sparse vegetation. The experiment was conducted for 68 nights in four phases. The first phase (pre-intervention phase) involved mosquito collections as described in the main manuscript, but without any ATSBs involved, and lasted 15 consecutive nights. In the second phase, ATSBs containing 1% borate were introduced into one densely-vegetated chamber, one medium vegetation chamber and one sparsely-vegetated chamber (five ATSBs were used per chamber, each suspended around the eave space). The other chambers were left without ATSBs to constitute contemporaneous controls (one densely-vegetated chamber, one sparsely vegetated chamber and one bare chamber).

Mosquito collections with HLC, CDC light traps and Prokopack aspirators continued as for 22 consecutive nights as described in the main manuscript. The third phase was similar to the first phase since the ATSBs were removed from all chambers, and the mosquito collections continued for another 11 nights. In the fourth phase, for another six nights, ATSBs were added in all chambers so that there were no contemporaneous controls. The fourth phase was conducted for 20 nights consecutively and was the reverse of the second phase in that the chambers that had been assigned ATSBs became controls while those that had been controls now received ATSBs.

Based on this configuration, there were therefore 26 nights during which all the chambers were controls, and 42 nights during which half of the chambers (one densely-vegetated, one sparsely vegetated, and one bare) were controls while the other half had ATSBs. Efficacy of the ATSBs was assessed as a percentage reduction of mosquitoes collected by the different traps.

The main observation was that the reduction of mosquito catches was generally similar across all chambers and did not appear to be impacted by vegetation cover. The efficacy of ATSBs against outdoor-biting mosquitoes (as caught by HLC) was 44%, 43% and 49% in chambers with sparse, medium and dense vegetation covers respectively (Figure S1). Against indoor-biting mosquitoes (as represented by CDC-light trap catches), the efficacy of ATSBs was 27%, 23% and 27% in chambers with sparse, medium and dense vegetation covers respectively (Figure S1). Similarly low or modest levels of efficacy were observed against resting mosquitoes collected either indoors or outdoors.


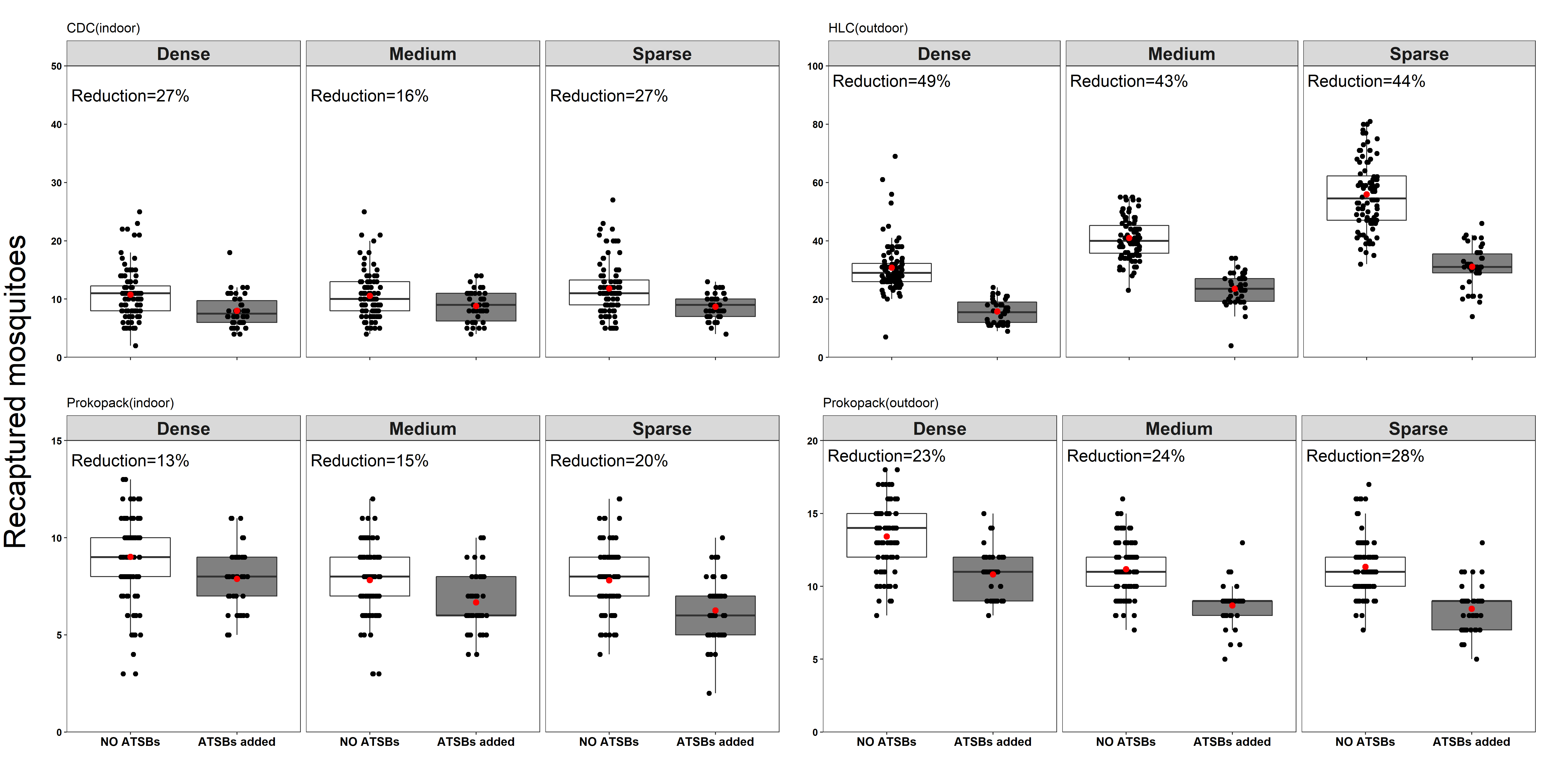


**Figure S1:** Efficacy of ATSBs with 1% boric acid against laboratory reared *Anopheles arabiensis* mosquitoes inside semi-field chambers with dense, medium and sparse vegetation covers.

**Experiment 3: Comparative Evaluation of the efficacy of ATSBs (active ingredient: 1% boric acid) in densely-vegetated, sparsely-vegetated and bare chambers**

Six semi-field chambers were used, two with dense vegetation, two with sparse vegetation, and another two with no vegetation. The experiment was conducted for 40 nights in six phases

The first phase (pre-intervention phase) involved mosquito collections as described above, but without any ATSBs involved, and lasted 5 consecutive nights. In the second phase, ATSBs containing 1% borate were introduced into one densely-vegetated chamber, one sparsely vegetated chamber and one bare chamber (five ATSBs per chamber, each suspended around the eave space). The other chambers were left without ATSBs to constitute contemporaneous controls (one densely-vegetated chamber, one sparsely vegetated chamber and one bare chamber). Mosquito collections with HLC, CDC light traps and Prokopack aspirators continued as above for 10 consecutive nights. The third phase was similar to the first phase since the ATSBs were removed from all chambers, and the mosquito collections continued for another five nights. In the fourth phase, for another five nights, ATSBs were added in all chambers so that there were no contemporaneous controls. The fifth phase was conducted for 10 nights consecutively and was the reverse of the second phase in that the chambers that had been assigned ATSBs became controls while those that had been controls now received ATSBs. In the final phase (sixth phase), the ATSBs were returned to all the chambers and the experiment continued for another five nights consecutively.

Based on this configuration, there were therefore 10 nights during which all the chambers were controls, 10 nights during which all the chambers had ATSBs and 20 nights during which half of the chambers (one densely-vegetated, one sparsely vegetated, and one bare) were controls while the other half had ATSBs. Efficacy of the ATSBs was assessed as a percentage reduction of mosquitoes collected by the different traps.

The main observation was that the reduction of mosquito catches was generally low across all chambers and did not appear to be impacted by vegetation cover except for mosquitoes collected using the CDC-light traps. The efficacy of ATSBs against outdoor-biting mosquitoes (as caught by HLC) was 19%, 23% and 27% in chambers with no vegetation, sparse vegetation and dense vegetation covers respectively (Figure S2). Against indoor-biting mosquitoes (as represented by CDC-light trap catches), the efficacy of ATSBs was 31%, 18% and 13% in chambers with no vegetation, sparse vegetation and dense vegetation covers respectively (Figure S1). We also observed either low or no protection against resting mosquitoes collected either indoors or outdoors.


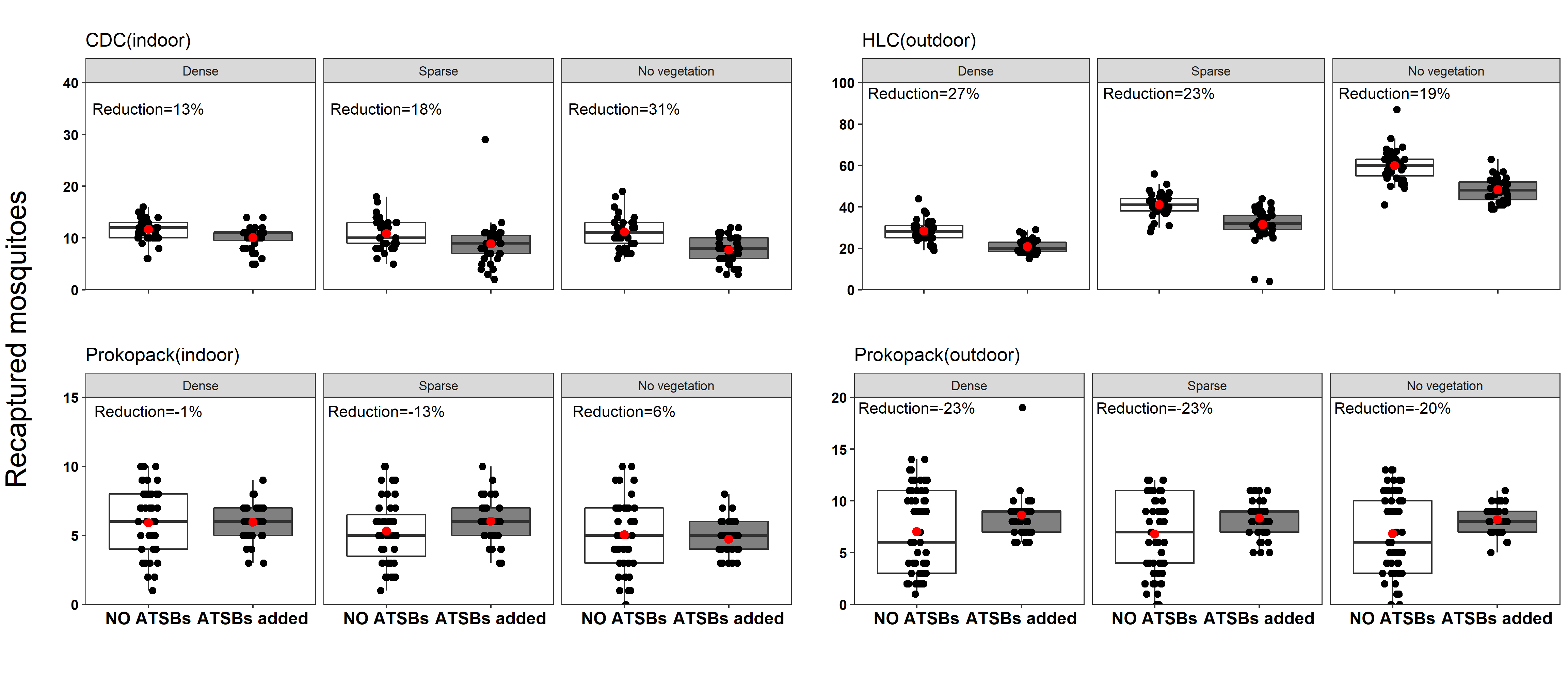


**Figure S2:** Efficacy of ATSBs with 1% boric acid against laboratory reared *Anopheles arabiensis* mosquitoes inside semi-field chambers with dense, sparse and no vegetation.

**Interpretation and decision relevant to the main study**

Following the three experiments above, a decision was made that subsequent studies (as reported in the main manuscript) would be done using ATSBs with 2% boric acid so as to maximize efficacy of the devices. In addition, to clarify any possible effects of vegetation cover, a further decision was made to discontinue the evaluation of medium vegetation, and instead only compare the ATSBs in densely-vegetated, sparsely-vegetate and non-vegetated chambers, as described in the main manuscript
